# Melatonin supplementation enhances browning suppression and improves transformation efficiency and regeneration of transgenic rough lemon plants (*Citrus × jambhiri*)

**DOI:** 10.1371/journal.pone.0294318

**Published:** 2024-03-06

**Authors:** Lamiaa M. Mahmoud, Nabil Killiny, Manjul Dutt

**Affiliations:** 1 Department of Horticultural Science, Citrus Research and Education Center, University of Florida, Lake Alfred, Florida, United States of America; 2 Department of Plant Pathology, Citrus Research and Education Center, IFAS, University of Florida, Lake Alfred, Florida, United States of America; Bahauddin Zakariya University, PAKISTAN

## Abstract

Enzymatic browning poses a significant challenge that limits in vitro propagation and genetic transformation of plant tissues. This research focuses on investigating how adding antioxidant substances can suppress browning, leading to improved efficiency in transforming plant tissues using *Agrobacterium* and subsequent plant regeneration from rough lemon (Citrus × jambhiri). When epicotyl segments of rough lemon were exposed to *Agrobacterium*, they displayed excessive browning and tissue decay. This was notably different from the ‘Hamlin’ explants, which did not exhibit the same issue. The regeneration process failed completely in rough lemon explants, and they accumulated high levels of total phenolic compounds (TPC) and polyphenol oxidase (PPO), which contribute to browning. To overcome these challenges, several antioxidant and osmoprotectant compounds, including lipoic acid, melatonin, glycine betaine, and proline were added to the tissue culture medium to reduce the oxidation of phenolic compounds and mitigate browning. Treating epicotyl segments with 100 or 200 μM melatonin led to a significant reduction in browning and phenolic compound accumulation. This resulted in enhanced shoot regeneration, increased transformation efficiency, and reduced tissue decay. Importantly, melatonin supplementation effectively lowered the levels of TPC and PPO in the cultured explants. Molecular and physiological analyses also confirmed the successful overexpression of the *CcNHX1* transcription factor, which plays a key role in imparting tolerance to salinity stress. This study emphasizes the noteworthy impact of supplementing antioxidants in achieving successful genetic transformation and plant regeneration in rough lemon. These findings provide valuable insights for developing strategies to address enzymatic browning and enhance the effectiveness of plant tissue culture and genetic engineering methods with potential applications across diverse plant species.

## Introduction

Citrus is a globally significant commodity cultivated in tropical and subtropical regions [1]. However, citrus production faces numerous challenges that hinder the maintenance and improvement of yield, particularly due to susceptibility to abiotic and biotic stressors. Conventional breeding methods have been employed successfully to enhance citrus germplasm, but the process is time-consuming and difficult due to various factors such as heterozygosity, sexual incompatibility, polyembryony, parthenocarpy, juvenility, and limited natural sources [1, 2].

Genetic engineering through *Agrobacterium*-mediated transformation has emerged as a valuable tool for enhancing the gene pool of dicotyledonous crops [3]. Successful transformation has been achieved in various fruit crops, including citrus, allowing the insertion of single or multiple genes to confer desirable traits absent in cultivated citrus varieties. For instance, the insertion of the *Arabidopsis thaliana NPR1* gene has provided sustained tolerance to citrus greening disease and salinity stress in sweet orange cultivars [4, 5]. Genetically modified citrus cultivars expressing anthocyanins have been developed to produce potentially higher nutrient and antioxidant contents [6–8]. Recent research has shown that overexpression of the tobacco salicylic acid binding protein 2 (SABP2) enhances tolerance against Huanglongbing disease in transgenic citrus [9]. Therefore, genetic engineering through citrus transformation presents an alternative approach for incorporating desirable traits into citrus genotypes.

Despite significant progress in citrus transformation, the citrus genus is not a natural host for *Agrobacterium tumefaciens*, and various citrus species exhibit low transformation efficiency, posing challenges for *Agrobacterium*-mediated transformation [10, 11]. Some lemon cultivars, in particular, have been reported to be difficult to transform using *Agrobacterium* cells [12], indicating the need for further development and optimization to improve transformation efficiency.

In fresh-cut fruits, vegetables, and tissue culture, the synthesis and accumulation of wound-induced phenolic compounds, which contribute to tissue browning, have been extensively studied [13, 14]. After tissue wounding, phenylalanine ammonia-lyase (PAL) is induced, leading to enhanced synthesis and accumulation of phenolic compounds [15, 16]. Excess phenolic compounds can be enzymatically oxidized to form quinones, as they undergo a chemical transformation that involves the removal of electrons and the creation of a quinone structure, which contributes to various biochemical processes, including enzymatic browning, discoloration, and degradation of plant tissues [17, 18]. Cellular metabolism produces free radicals or reactive oxygen species (ROS) as byproducts, and under stress conditions, excessive accumulation of such compounds can result in oxidative stress [19]. Several approaches have been employed to overcome the browning reactions caused by accumulated phenolic compounds, including the application of antioxidants to exclude oxygen or inhibit the activity of enzymes involved in browning. Antioxidants play a crucial role as non-enzymatic scavengers of ROS. They have been shown to effectively delay or even prevent the oxidation of various biomolecules, including proteins, lipids, carbohydrates, and DNA, particularly when present in low concentrations. By neutralizing ROS, antioxidants help maintain cellular homeostasis and protect biomolecules from oxidative damage, which can have detrimental effects on cellular function and overall health [20].

Alpha-lipoic acid is a short-chain fatty acid that is present in various organisms and has gained attention for its antioxidant and therapeutic properties [21, 22]. Studies have shown that lipoic acid can enhance photosynthetic performance, regulate ion homeostasis, mitigate lipid peroxidation, and induce antioxidant systems in crops under osmotic stress [21, 23, 24]. Additionally, the inclusion of lipoic acid in culture media during *Agrobacterium*-mediated transformation has significantly improved the transformation efficiency of crops, even for previously difficult-to-transform genotypes [19].

Melatonin, a naturally occurring indole-like hormone, acts as an antioxidant and protects against ROS [25]. Exogenous melatonin treatment has been shown to enhance plant responses to environmental factors [26–29] and disease [30–33]. In previous studies, melatonin was utilized to enhance the stable transformation efficiency in tomato (MicroTom cultivar), soybean and banana by mitigating cell death, controlled surface shrinkage and improving the attachment of *Agrobacterium* cells to the plant cell surface [34, 35] Glycine betaine is a compatible solute synthesized in chloroplasts that plays a crucial role in cellular defense and protects proteins, enzymes, and membranes against stress-induced damage [36]. It stabilizes macromolecules and protein structures and maintains ROS scavenging capacity [37, 38]. Proline, an osmoprotectant, serves multiple functions in plant cells [39]. It acts as a potent nonenzymatic antioxidant and prevents oxidative damage by stabilizing DNA, membranes, and protein complexes, and regulates intracellular redox potential and energy storage [39–41].

Rough lemon is a citrus rootstock variety that has enhanced tolerance to Huanglongbing (HLB) [42], an often fatal bacterial disease affecting citrus [43]. Rough lemon is also important in several parts of the world as a valuable rootstock but is very susceptible to salinity induced abiotic stress and root rot caused by *Phytophthora* spp. However, unlike ‘Hamlin’ sweet orange, a leading early season sweet orange, this cultivar is difficult to transform using *Agrobacterium* and there are few reports of its successful genetic transformation [44]. Given the importance of rough lemon in several parts of the world, this study was undertaken to enhance the efficiency of *Agrobacterium*-mediated transformation and regeneration. We hypothesized that supplementing the tissue culture medium with antioxidant compounds has the potential to reduce enzymatic browning by decreasing the levels of total phenolic compounds (TPC) and polyphenol oxidase (PPO) in rough lemon. Consequently, this could enhance the efficiency of genetic transformation and subsequent plant regeneration. We evaluated the effects of antioxidant compounds (lipoic acid and melatonin) and osmoprotectants (glycine betaine and proline) to effectively reduce phenol oxidation and mitigate browning. Subsequently we produced transgenic plants with this improved protocol and evaluated their tolerance to enhanced levels of salt.

## Methods

### DNA construct

The binary vector pC2300-NHX1-E was utilized as the basis for all overexpression experiments while the binary vector pC2300-NHX1-EGFP was used for confocal studies. The pC2300-NHX1-E vector consists of the *CcNHX1* gene (NCBI accession: OR047644; identified from the Clementine genome) driven by a CaMV 35S promoter. The EGFP gene was driven by a CsVMV promoter, along with the *npt*II gene driven by the NOS promoter. The pC2300-NHX1-EGFP vector consisted of the *CcNHX1* gene fused in frame to the EFGP gene and driven by the 35S promoter (NCBI accession: OR047645). The construct also contained the *npt*II gene driven by the NOS promoter. All plasmid DNA was transformed into the *A*. *tumefaciens* AGL1 strain.

### *Agrobacterium*-mediated transformation and antioxidant supplementation to tissue culture media

Rough lemon and ‘Hamlin’ seeds were germinated as previously described [10] and 1 cm long epicotyl segments were used for all transformation experiments. A single colony of the *A*. *tumefaciens* was cultured in liquid YEP medium containing kanamycin (100 mg/L) and rifampicin (20 mg/L) at 26°C and 185 rpm for 2 d. Subsequently, 2 mL of a vigorously growing *Agrobacterium* culture, initiated the previous night, was added to 48 mL of YEP medium containing appropriate antibiotics. The cells were cultured for 3 h, followed by centrifugation at 4000 × *g* for 10 min at 25°C. The resulting pellet was resuspended in liquid co-cultivation medium (CM) [10] to achieve OD600 values of 0.2, which were used in all experiments. The plant regeneration media (RM) was supplemented with antioxidant compounds (lipoic acid at concentrations of 50, 100, and 200 μM and melatonin at concentrations of 50, 100, and 200 μM) or osmoprotectants (glycine betaine at concentrations of 20, 40, and 100 mM, and proline at concentrations of 20, 40, and 100 mM). Shoots were observed for EGFP fluorescence using a Zeiss Scope A1 fluorescence microscope (Carl Zeiss Microscopy, Gottingen, Germany) equipped with a FITC filter. Images were captured with Leica’s LASX software (Carl Zeiss Microscopy GmbH, Göttingen, Germany) coupled to a Zeiss Axio Cam ICc1.

Regeneration and transformation efficiency percentages were calculated after a 45-day period. Regeneration percentage was determined using the formula: [Number of regenerated shoots / Total number of segments per plate] × 100. Transformation efficiency percentage was calculated as: [Number of transformed shoots / Total number of regenerated shoots per plate] × 100.

### Confocal microscopy

Confocal microscopy was utilized to examine the expression and localization of *CcNHX1*, which was fused with enhanced green fluorescent protein (*egfp*) coding sequence (*CcNHX1-egfp*) and driven by the 35S promoter. The binary plasmid construct containing *CcNHX1-egfp* was introduced into *A*. *tumefaciens* strain EHA105. A single colony of EHA105 containing the construct was cultured in Luria-Bertani (LB) broth supplemented with 25 mg/L rifampicin and 100 mg/L kanamycin. After overnight incubation at 28°C, the bacterial culture was centrifuged, and the pellet was resuspended in an infiltration buffer containing 2-(N-morpholino) ethanesulfonic acid (MES) and MgCl_2_ at 10 mM; pH 5.85, supplemented with 200 μm acetosyringone. The bacterial suspension was incubated at the room temperature for 4 h, then infiltrated into the fully expanded leaves of *Nicotiana benthamiana* plants using a 1 mL needleless syringe [45]. Confocal imaging was performed on samples obtained three days after infiltration using a Leica Microsystems confocal microscope (Leica Microsystems Inc., Buffalo Grove, IL, USA).

### Determination of total phenolic compounds and polyphenol oxidase (PPO)

Fresh leaf samples weighing 100 mg were extracted with 80% ethanol according to Singleton and Rossi [46]. 100 μL of Folin reagent (1:10) was mixed with the extract, vortexed, and incubated for 5 min at room temperature. Then the reaction was induced by adding 300 mL of 20% sodium carbonate (Na_2_CO_3_) to the mixture, and the tubes were incubated in the dark for 1 h. The absorbance of the reaction mixture was measured at 765 nm. A standard curve was created using standard solutions of gallic acid (0–600 ppm). The phenolic content was expressed as milligrams of gallic acid per 100 milligrams of fresh weight tissue.

The activity of polyphenol oxidase (PPO) was estimated as reported by [47]. Stem tissues were homogenized in a buffer solution containing 0.05 M Tris-HCl buffer solution (pH 7.2) containing 0.4 M sorbitol solution and 0.01 M NaCl. The homogenate was centrifuged at 20,000 rpm; 4°C for 5 min, and the resulting supernatant was used as the enzyme extract. A substrate mixture composed of 2.5 mL of 0.1 M phosphate buffer (pH 6.5) and 0.3 mL of 0.01 M catechol solution was prepared, and the quinone production was monitored spectrophotometrically at 420 nm. PPO is expressed as ng.g^-1^FW.

### *CcNHX1* transgene assessment

Genomic DNA was extracted from the regenerated plants using the GeneJET Plant Genomic DNA Purification Mini Kit (Thermo Fisher Scientific, Waltham, MA) according to the manufacturer’s instructions. The presence of the *CcNHX1* transgenes in the potential plants was verified through PCR analysis. PCR was conducted in a thermal cycler (C1000; Bio-Rad Laboratories, Hercules, CA) using GoTaq Green Master PCR Mix (Promega Corp, Madison WI). The forward primer was designed to be within the CcNHX1 gene while the reverse primer was obtained from the 35S-3’ terminator sequence (Table 1). The plants that exhibited positive PCR results were further propagated, and two specific lines were chosen for subsequent gene expression and salt stress experiments.

**Table 1:** List of the primer sequences used in confirmation of DNA integration and SYBR green based real-time PCR assay.

GenBank accession number	Description (gene symbol)	Primer name	Primer sequences (5’—3’)
OR047644	*CcNHX1*	NHX1-F	CGGACTGCTCAGTGCTTATATT
		35s-R	GCTCAACACATGAGCGAAAC
OR047644	*CcNHX1*	N-SYBR-F	CGGACTGCTCAGTGCTTATATT
		N-SYBR-R	CAGCCAGCATATACGAAAGGT
XM_006464503.3	β-actin	A-SYBR-F	GCTGCCTGATGGCCAGATC
		A-SYBR-F	AGTTGTAGGTAGTCTCATGAA

RNA was isolated from finely ground leaf tissues of the selected two lines using a commercially available RNA extraction kit (Directzol^™^ RNA Miniprep kit; Zymo Research, Irvine, CA, USA). The isolated RNA samples were then subjected to qPCR analysis using the PowerUp^™^ SYBR^™^ Green Master Mix (Thermo Fisher Scientific Inc. Waltham, MA, USA). The obtained Ct values were compared to those of non-transgenic rough lemon plants. To determine the relative expression of the target gene, the 2^^-ΔΔCT^ method [48] was employed, with the actin gene used as an internal housekeeping control. The primer sequences for the evaluated genes can be found in Table 1.

### Salt stress assay in transgenic *CcNHX1* lines

Leaf disks (1 cm in diameter) from 2 year old transgenic lines were prepared from leaves of wild-type and transgenic plants according to Sun, Wang [49]. These leaf disks were floated on solutions with 0, 100, 200 and 300 mM NaCl concentrations for 72 h. The phenotype of the leaf disks was photographed, and the total chlorophyll content was measured following the salt treatment. Chlorophyll content was determined by macerating the leaf disks in 100% methanol and measuring absorbance values at 665 nm 653 and 470 nm wavelengths. Chlorophyll a, chlorophyll b and total chlorophyll were estimated according to Lichtenthaler and Wellburn [50].

### Statistical analysis

A statistical analysis was carried out using a one-way analysis of variance (ANOVA) in a completely randomized design with ten replicates per treatment. A statistical analysis for salt stress assay in transgenic *CcNHX1* lines was carried out using a two-way analysis of variance (ANOVA) in a factorial-based complete randomized design with five salt levels (0,50, 100, 200 and 300 mM NaCl) and three lines (control wild type, *CcNHX1*-Line 1, and *CcNHX1*-Line 2) with nine replicates. To identify significant differences between treatments, a post hoc analysis was conducted using the Tukey-Kramer HSD test. The statistical analyses were performed using JMP Pro version 16, and significance was determined based on *p*-values less than 0.05.

## Results

### Regeneration from rough lemon and ‘Hamlin’ explants

**Fig 1:**
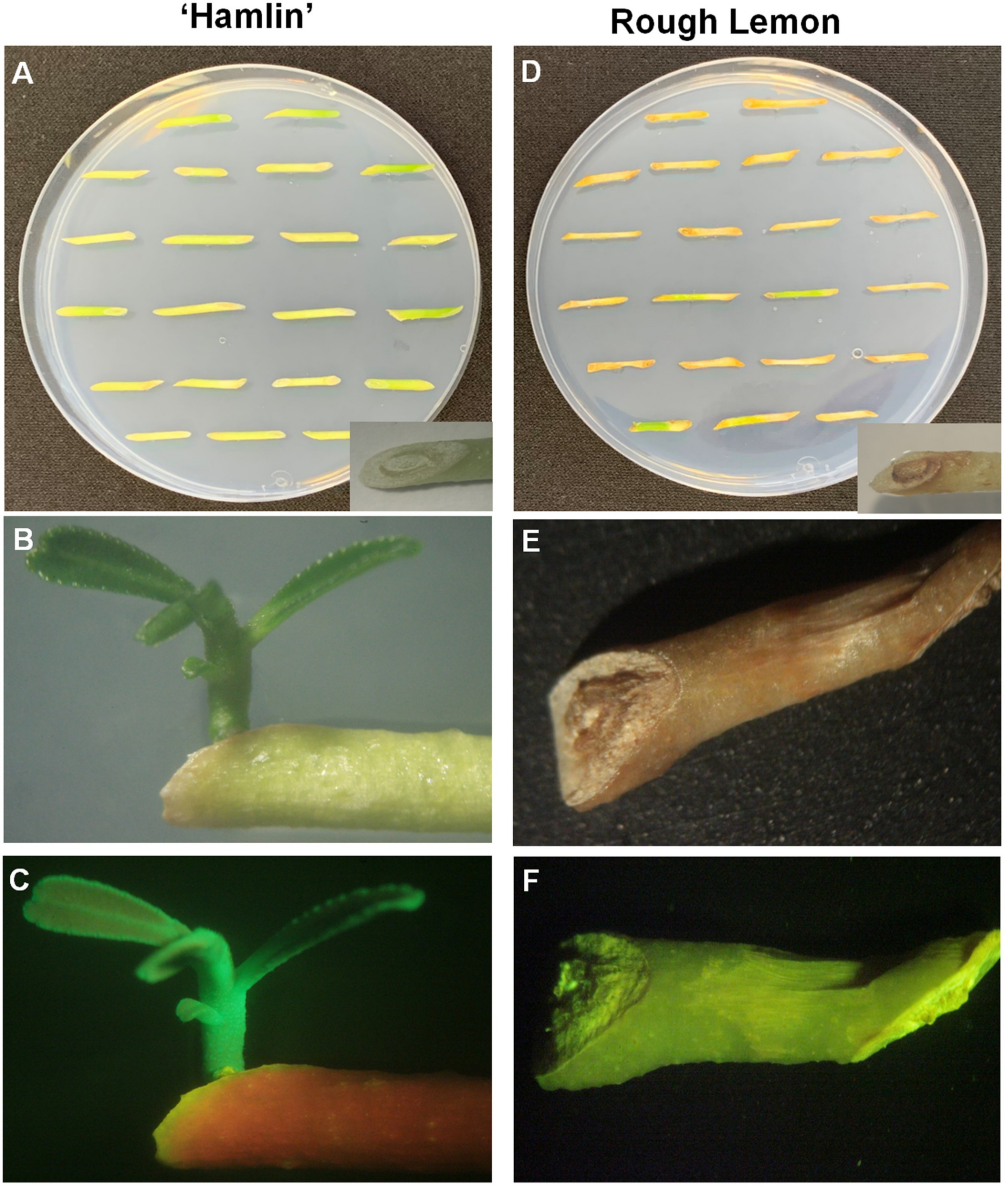
Images show ‘Hamlin’ and rough lemon tissues following *Agrobacterium*-mediated transformation and 45 days incubation at the regeneration media. A comparison between the morphology of rough lemon stems (A) and ‘Hamlin’ (D) after one week of cultivation in regeneration media. B and E the same ‘Hamlin’ stem exhibiting EGFP expression under an epi-fluorescence stereomicroscope. (C and F) the same rough lemon stem exhibiting EGFP expression under an epi-fluorescence stereomicroscope.

**Fig 2:**
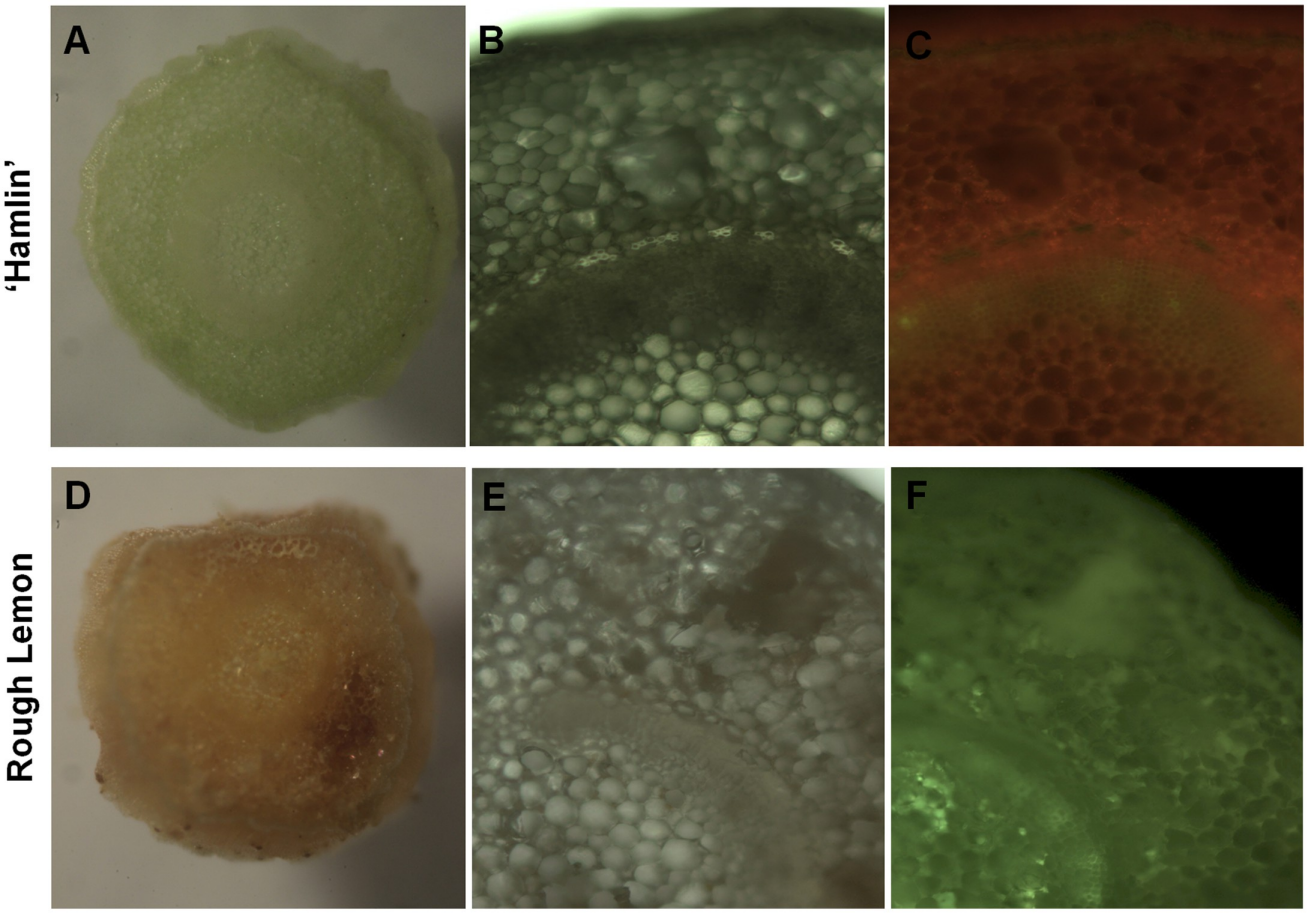
Cross-section of ‘Hamlin’ (A) and rough lemon (D) tissues following *Agrobacterium*-mediated transformation and 45 days incubation at the regeneration media. (B and C) the same ‘Hamlin’ stem section exhibiting under an epi-fluorescence stereomicroscope. (E and F) the same rough lemon stem section exhibiting under an epi-fluorescence stereomicroscope.

‘Hamlin’ sweet orange was used as the positive control in this study due to its ability to easily transform using *Agrobacterium* [10]. Following *Agrobacterium*-mediated transformation of rough lemon and ‘Hamlin’ explants, significant differences in their response to regeneration medium (RM) were observed (Fig 1). ‘Hamlin’ explants remained green and successfully regenerated and produced healthy transgenic lines after 30 days of culture. Microscopic examination of ‘Hamlin’ tissues confirmed their overall health and absence of browning. In contrast, rough lemon explants exhibited severe browning and failed to regenerate or produce any plants when placed in regular plant regeneration medium without the addition of any supplements. Microscopic sections of rough lemon explants further confirmed the presence of unhealthy tissues and the accumulation of browning pigments on the tissue (Fig 2). These results highlight the contrasting behavior of rough lemon and ‘Hamlin’ explants, emphasizing the challenge of tissue browning in rough lemon and its detrimental effect on regeneration and plant production.

### Effect of antioxidants treatment on regeneration improvement and transformation efficiency in rough lemon segments

**Fig 3:**
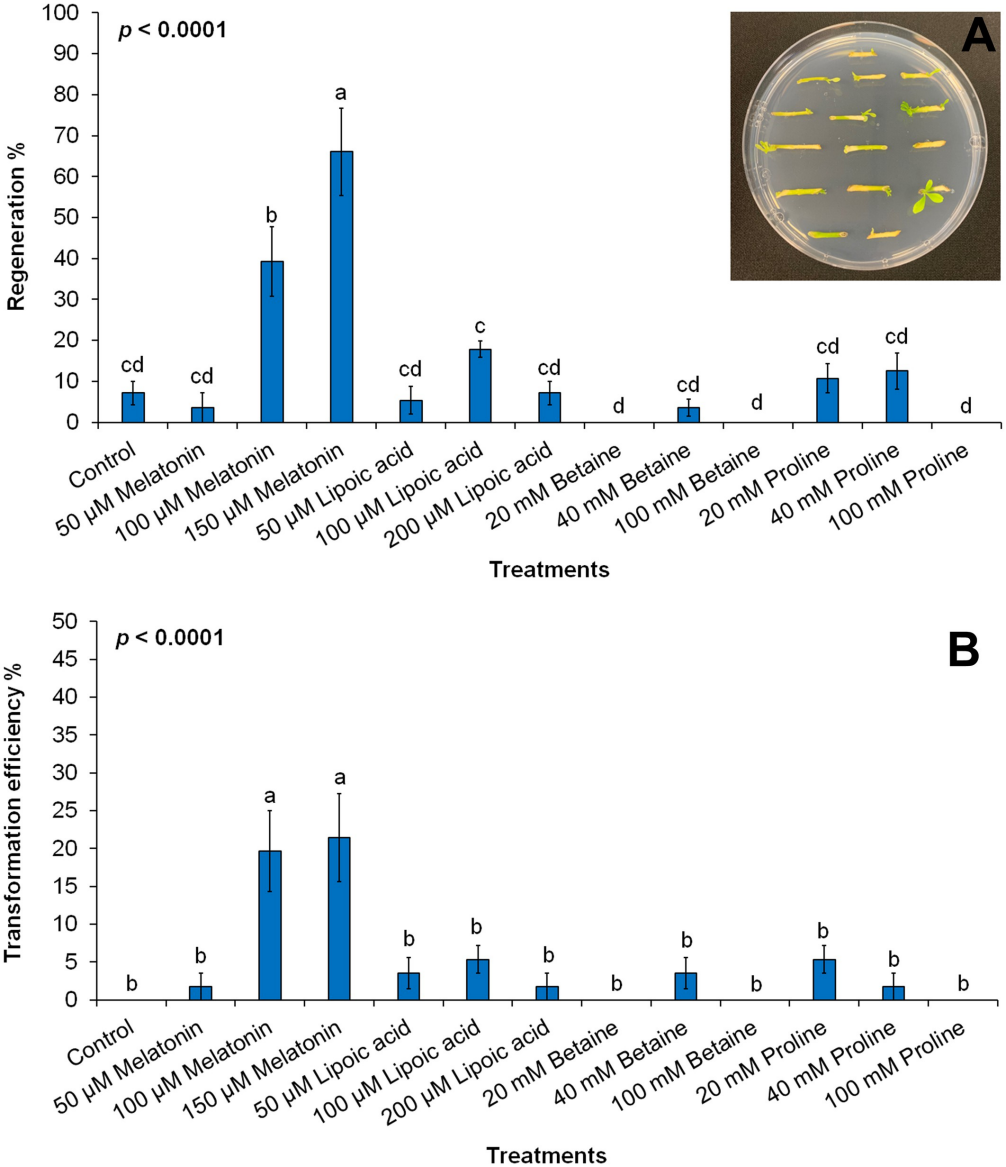
Regeneration improvement (A) and transformation efficiency (B) in rough lemon tissues following antioxidants supplementation to regeneration media. (Inset) Generation of rough lemon stems in regeneration media (RM) supplemented with Melatonin at 200 μM.

**Fig 4:**
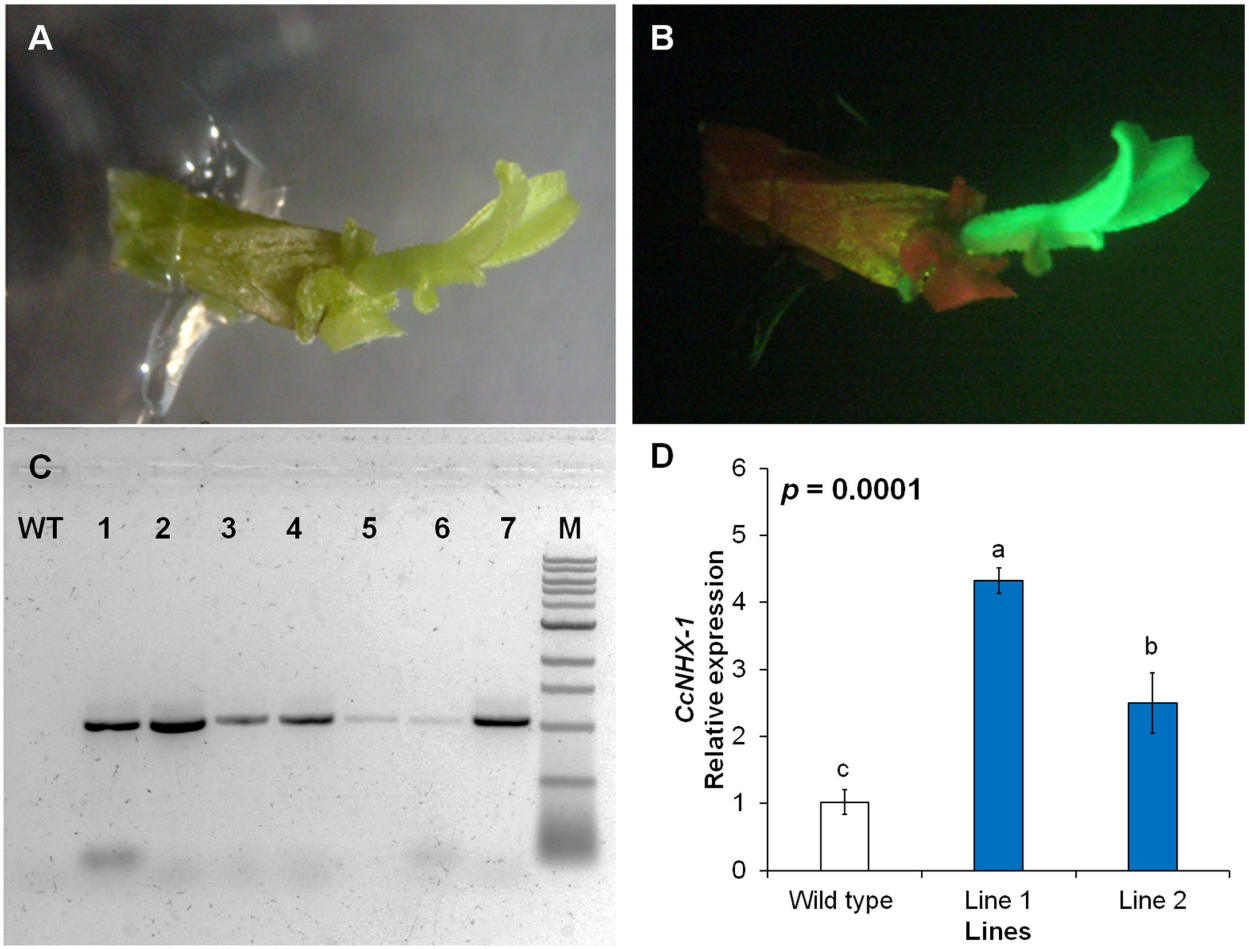
Integration and expression of *CcNHX1* (Na^+^/H^+^ exchanger) gene in rough lemon plants. (A) Bright field of rough lemon shoots after regeneration targeting *CcNHX1* overexpression, (B) GFP-expressed shoot, (C) Amplification products obtained from PCR of transgenic rough lemon genomic DNA with 1000 bp fragment of the *CcNHX1* gene, 1 kb marker. (D) Relative expression of *CcNHX1* gene in transgenic lines compared to control wild type.

Subsequently, we investigated the role of supplemental antioxidant addition to the RM on the regeneration improvement and transformation efficiency of rough lemon epicotyl segments. The expression of the transgene was assessed by monitoring the green fluorescence protein (GFP) expression in the leaves. The results indicated that the supplemental antioxidant significantly enhanced plant regeneration and transformation efficiency in rough lemon segments (Fig 3). The optimal conditions for plant regeneration and transformation were achieved when the epicotyl segments were cultured on a regeneration medium supplemented with 100 or 200 μM melatonin. The regeneration percentage was recorded as 39.2% and 66% in the media supplemented with 100 μM and 200 μM, respectively. However, there was no significant difference compared to the control regeneration media when the 50 μM melatonin was supplied to the media. Similarly, when comparing the other antioxidants to the control, no observable differences were recorded. The observed improvement in regeneration and transformation efficiency suggests that melatonin treatment effectively promoted the growth and development of rough lemon tissues, leading to successful transgene expression (Figs 3 and 4).

### Generation of transgenic *CcNHX1* lines and salt stress tolerance

Following transformation and successful regeneration, the expression and functionality of the introduced transgenes were investigated. Transgenic lines of rough lemon were initially screened based on the presence of green fluorescent protein (GFP) as a marker for successful gene integration (Fig 4A and 4B). Subsequently, a subset of seven plantlets expressing the enhanced green fluorescent protein (EGFP) was randomly selected for further analysis. Genomic DNA (gDNA) was extracted from these plantlets, and PCR was conducted to determine the presence of the *CcNHX1* gene. The PCR analysis of the gDNA samples demonstrated that eight plant lines tested positive for the presence of the *CcNHX1* gene (Fig 4C). Gene expression analysis was conducted to confirm the integration of the target gene in two selected transgenic lines (Fig 4D). Following the confirmation of gene integration, five transgenic lines were propagated for further evaluation. Among them, two positive lines were selected for subsequent experiments.

### Salt stress assessment

Following the confirmation of Integration of *CcNHX1* (Na^+^/H^+^ exchanger) gene in rough lemon plants, two positive lines were selected for subsequent experiments. We studied the performance of these transgenic lines under NaCl stress conditions compared to non-transgenic controls. In the leaf disk floating assay, the non-transgenic control leaves displayed visible damage when exposed to a high concentration of 300 mM NaCl, whereas the transgenic leaves remained healthy. Furthermore, a significant reduction in chlorophyll content was observed in the non-transgenic control under the same NaCl stress condition (Table 2). This suggests that the transgenic lines exhibited enhanced salt tolerance compared to the non-transgenic controls, as evidenced by their improved physiological performance, and preserved chlorophyll content even under high salt stress. These results indicate the potential of the introduced gene in conferring salt tolerance to the transgenic rough lemon lines, highlighting the significance of this genetic modification in overcoming the detrimental effects of salt stress on plant growth and development.

**Table 2:** Content of chlorophyll pigments in leaf discs from wild type and transgenic rough lemon plants treated with different concentrations of NaCl.

Variables	NaCl treatments	WT	*CcNHX1*-LINE 1	*CcNHX1*-LINE 2
**Chlorophyll A (mg**^**−1**^ **g FW)**	CONTROL	8.27 ± 0.51^a A^	8.15 ± 0.09^a A^	8.38 ± 0.44^a A^
	50 mM NaCl	4.91 ± 0.78^a B^	5.00 ± 0.25^a B^	6.45 ± 1.42^a AB^
	100 mM NaCl	3.98 ± 0.07^b B^	4.74 ± 0.66^ab B^	6.39 ± 0.22^a AB^
	200 mM NaCl	1.91 ± 0.07^b C^	3.56 ± 0.52^a B^	3.87 ± 0.22^a BC^
	300 mM NaCl	1.47 ± 0.09^c C^	4.25 ± 0.23^a B^	3.30 ± 0.26^b C^
**Chlorophyll B (mg**^**−1**^ **g FW)**	CONTROL	2.29 ± 0.41^a A^	1.64 ± 0.23^a A^	1.39 ± 0.48^a A^
	50 mM NaCl	1.56 ± 0.57^a A^	1.64 ± 0.53^a A^	1.68 ± 0.40^a A^
	100 mM NaCl	1.69 ± 0.04^a A^	1.63 ± 0.60^a A^	0.66 ± 0.27^a A^
	200 mM NaCl	1.05 ± 0.10^a A^	1.42 ± 0.48^a A^	1.21 ± 0.01^a A^
	300 mM NaCl	1.67 ± 0.01^a A^	1.00 ± 0.14^b A^	1.19 ± 0.16^b A^
**Total chlorophyll (mg**^**−1**^ **g FW)**	CONTROL	10.57 ± 0.79^a A^	9.80 ± 0.19^a A^	9.78 ± 0.09^a A^
	50 mM NaCl	6.47 ± 0.21^a B^	6.65 ± 0.33^a B^	8.14 ± 0.86^a B^
	100 mM NaCl	5.67 ± 0.02^c B^	6.38 ± 0.05^b B^	7.06 ± 0.05^a B^
	200 mM NaCl	2.96 ± 0.02^b C^	4.99 ± 0.05^a C^	5.08 ± 0.09^a C^
	300 mM NaCl	3.14 ± 0.10^b C^	5.26 ± 0.09^a C^	4.50 ± 0.43^a C^

*Means followed by the same letter were not significantly different at (*p* < 0.05). Difference between lines (columns) at a particular treatment (row) is indicated by differing lowercase letters; mean separation between treatments (rows) in a particular line (column) is indicated by differing uppercase letters by Tukey’s honestly significant difference test (*p* ≤ 0.05). numbers represent means ± SE (n = 9).

### Changes of total phenolic and PPO activity in rough lemon tissues following antioxidants supplementation

**Fig 5:**
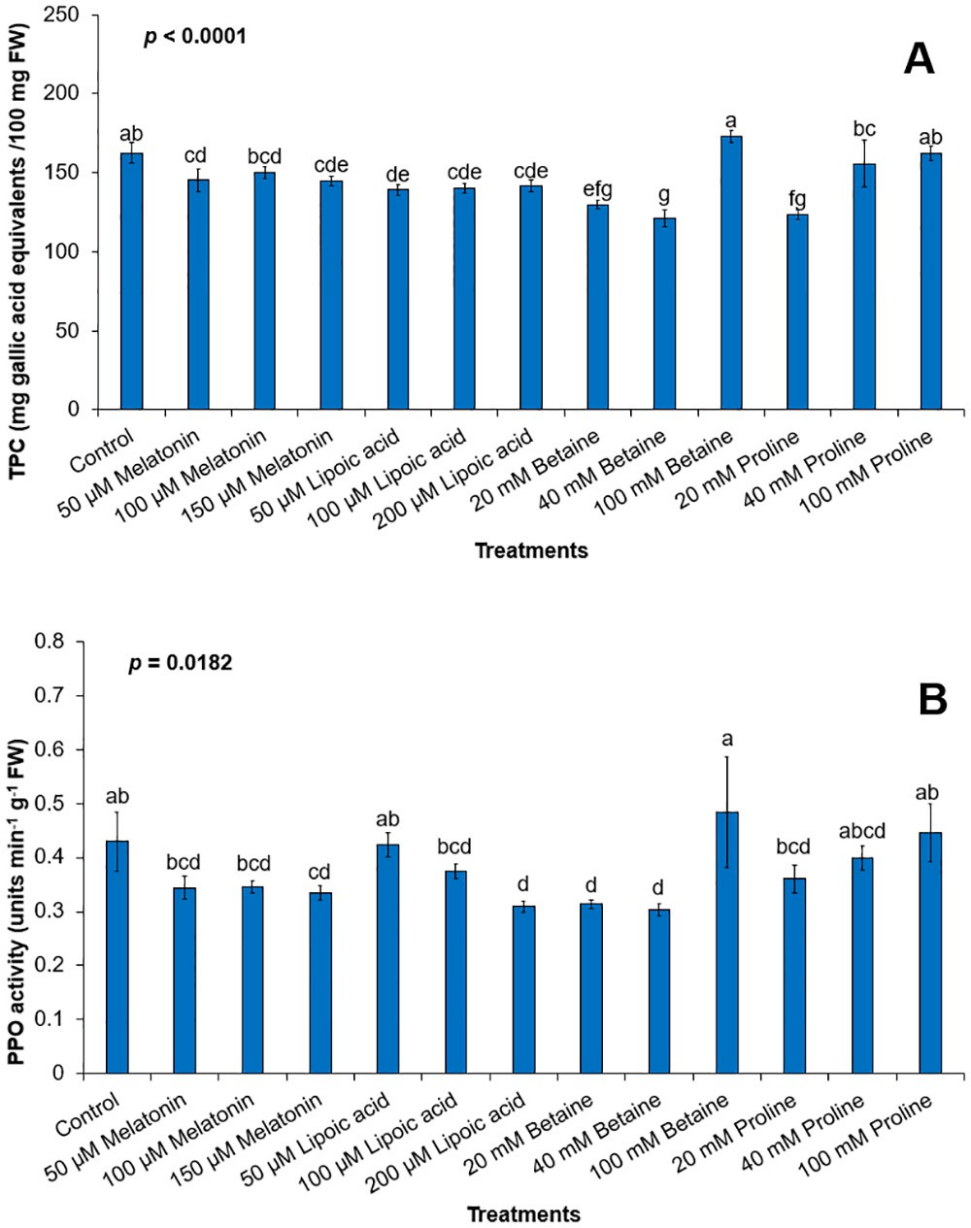
Total phenolic (A) and polyphenol oxidase activity (PPO) (B) in rough lemon tissues following antioxidants supplementation to regeneration media. TPC is expressed as mg gallic acid equivalents /100 mg FW and PPO is expressed as ng.g^-1^FW.

We hypothesized that the necrosis and tissue browning observed in cultured rough lemon explants, following injury and *A*. *tumefaciens* infection, were attributed to the activation of the endogenous defense response and subsequent induction of phenolic compounds due to the generation of ROS. Our results demonstrated a significant increase in TPC in the explants cultured in RM, indicating the induction of phenolic compounds as part of the defense response. We observed that antioxidant supplementation led to a reduction in TPC induction compared to the untreated explants (Fig 5). This suggests that the addition of antioxidants to the media effectively mitigated the activation of the defense response and subsequent phenolic compound accumulation in the explants. Furthermore, we observed a correlation between the decreased TPC levels and the activity of PPO. The overdose of betaine and proline induced the accumulation of TPC and PPO activity, potentially indicating a role for these compounds in promoting oxidative browning.

### Total phenolic compounds and PPO activity in ‘Hamlin’ and rough lemon tissues

**Fig 6:**
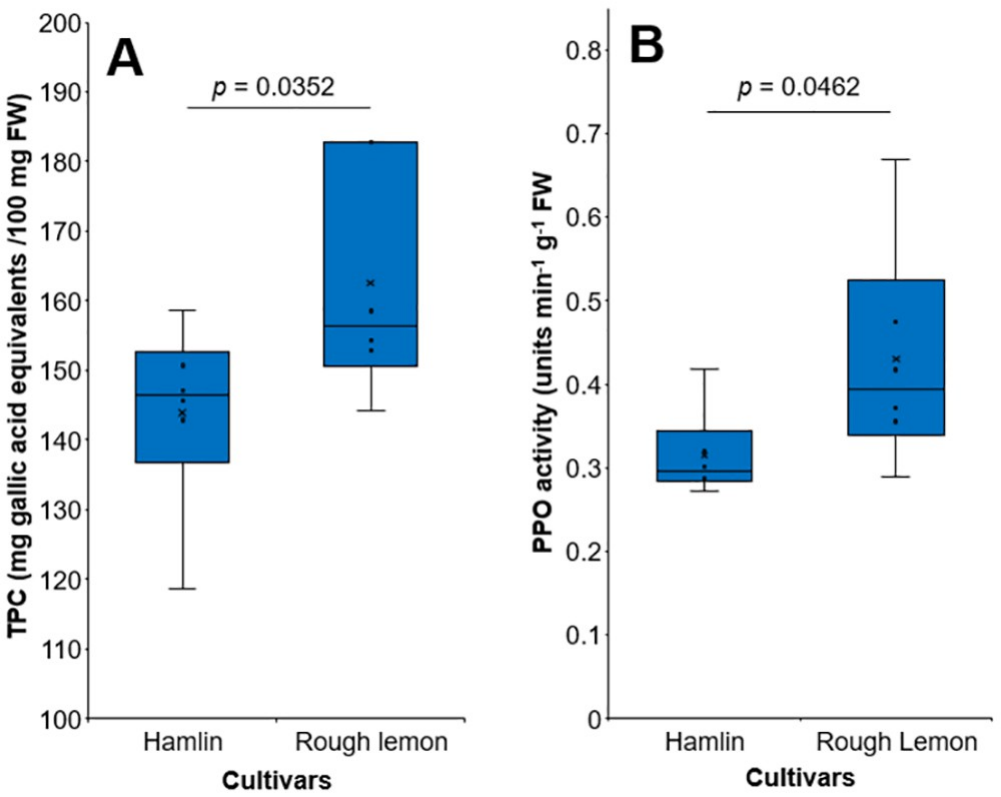
Total phenolic compounds (A) and Polyphenol oxidase activity (PPO) (B) in ‘Hamlin’ and rough lemon tissues.

A significant difference (*p* < 0.05) was observed when the two cultivars were compared (Fig 6). Rough lemon tissues exhibited a significant increase in TPC content compared to ‘Hamlin’ tissues, indicating a higher accumulation of phenolic compounds in the rough lemon. This observation was consistent with the increased activity of PPO in rough lemon tissues, suggesting a potential correlation between TPC levels and PPO activity in citrus tissues.

### CcNHX1 is localized in the cell membrane

**Fig 7:**
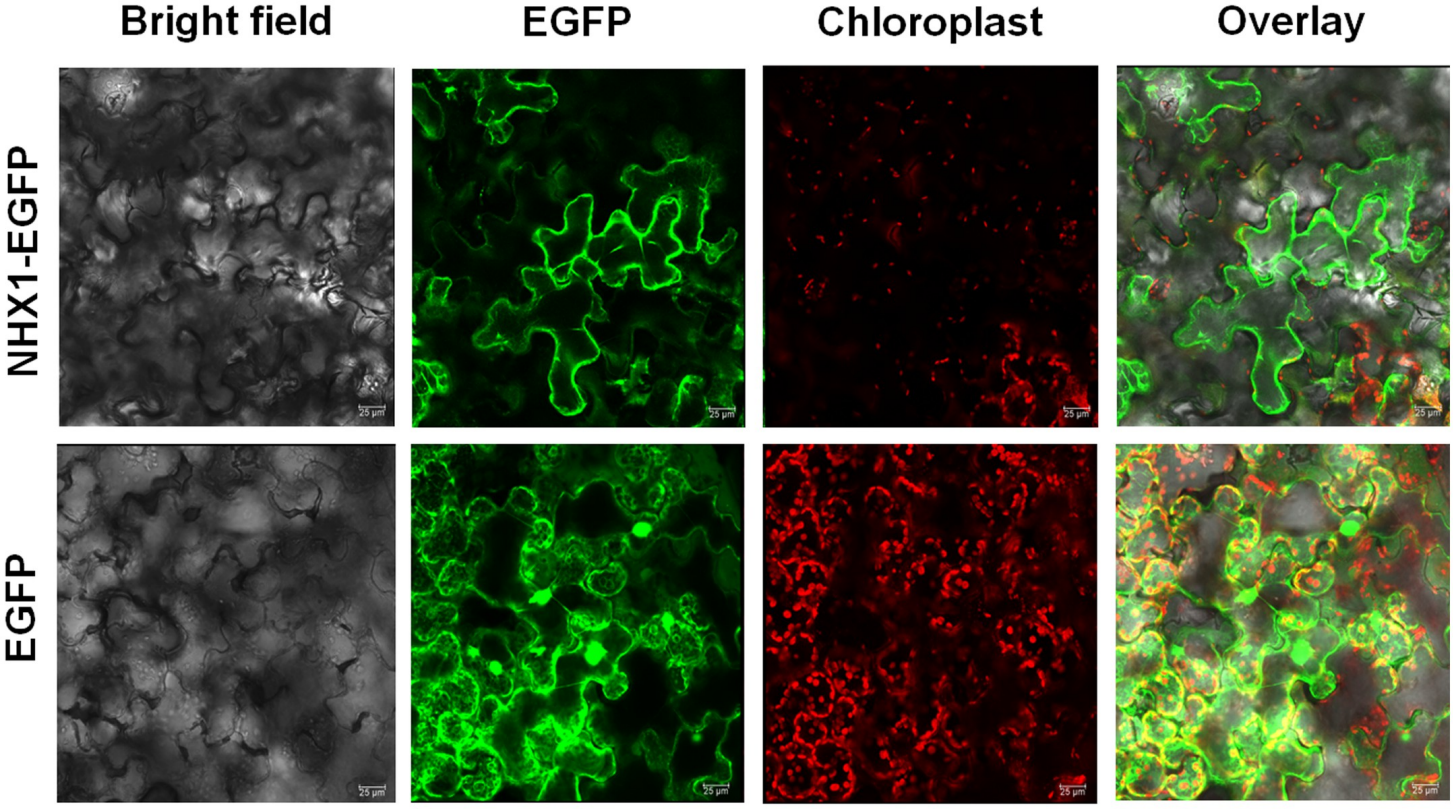
Confocal micrographs showing transient expression of NHX1-EGFP fusion protein. Confocal images showing micrographs of transmission white light (Bright field), EGFP, chloroplast, and the resultant overlay, respectively. The EGFP panels indicate cell membrane localization of NHX1 fused to EGFP under expression of the 35S promoter. EGFP on its own was used as the positive control.

The subcellular localization of the CcNHX1 protein was investigated in this study. We observed that the CcNHX1-EGFP fusion protein exhibited subcellular localization in the membrane, indicating its association with the cellular membranes (Fig 7). The fluorescence signal and localization pattern of the CcNHX1-EGFP fusion protein were comparable to those of the positive control, which expressed EGFP alone. These results suggest that the CcNHX1 protein is targeted to the cellular membrane, consistent with its proposed role in ion transport and membrane-associated functions. The subcellular localization of CcNHX1 provides valuable insights into its potential mechanisms of action and its involvement in cellular processes related to membrane dynamics and ion homeostasis.

## Discussion

Plants have developed defense and protection mechanisms that involve various cellular processes and hormone regulation to withstand harsh environmental conditions [51, 52]. Plant cells produce phenolic compounds in response to stressors as a defensive mechanism. These phenolic compounds play a significant role in protecting plants against pathogens, pests, and environmental stressors. However, excessive buildup of phenolic compounds can lead to the formation of quinones. o-Quinones, generated through the hydroxylation and oxidation of phenols, can interact with glutathione (GSH), depleting its levels and leading to oxidative stress [53]. Additionally, o-quinones can alkylate or oxidize cysteine residues in proteins, affecting their function and triggering signaling pathways [54]. o-Quinones can also form DNA adducts and oxidize DNA bases, potentially causing genotoxic effects [55]. These compounds exhibit various activities, including detoxification, chemoprevention, and toxicity, depending on their interactions with cellular components [56]. Additionally, the presence of quinones can result in severe tissue browning, which can negatively impact the overall health and appearance of the plant [57].

Plant tissue culture techniques necessitate physical damage, such as cuts and wounds, to the plant tissue [58]. This mechanical injury can trigger tissue browning, which disrupts the cellular integrity. The susceptibility to tissue browning may vary among different plant varieties or cultivars due to inherent genetic factors [59]. These genetic factors can impact the plant’s ability to respond to stress and maintain cellular integrity. In our investigation, we noticed distinct responses in rough lemon and ‘Hamlin’ explants concerning regeneration and browning after injury and *A*. *tumefaciens* infection.

Rough lemon explants exhibited severe browning and necrosis, unlike the response observed in ‘Hamlin’ explants. This disparity suggests the presence of specific genetic factors that contribute to this variation in their reactions. These genetic factors likely play a crucial role in regulating physiological and biochemical processes related to tissue browning and necrosis [60]. It is worth noting that rough lemon has been widely used as a rootstock in various regions due to its proven tolerance to HLB. However, this rootstock is highly susceptible to salinity-induced abiotic stress. When the soil has excessive salt levels, it can have detrimental effects on the growth and development of rough lemon plants, resulting in reduced productivity and potential damage.

Genetic transformation is a highly effective method to improve plants by incorporating favorable traits or altering existing ones [61]. However, this process of introducing foreign genes into an organism’s genome can lead to tissue damage [58]. The tissue damage can be attributed to various factors associated with the transformation process, such as physical injury, chemical stress, oxidative stress, and immune response 59. Notably, *Agrobacterium* infection has been observed to induce cell necrosis in numerous plant species, including both monocots and dicots like sugarcane, wheat, maize, grape, soybean, sorghum, tomato, and banana [62].

Due to the limitations associated with tissue damage during genetic transformation, the development of a new protocol became necessary to enhance biotechnological approaches for producing new clones that exhibit increased tolerance to stressors. Several studies have reported that the addition of antioxidants during the process of plant transformation can improve the efficiency of the transformation by preventing cell death [63, 64]. Antioxidant supplementation played a crucial role in mitigating tissue browning and improving regeneration and transformation efficiency in rough lemon segments. The optimal regeneration and transformation conditions were achieved when epicotyl pieces were cultured on a regeneration medium supplemented with 100 and 200 μM melatonin. In previous studies, a similar concentration of melatonin (100 μM) was employed to enhance the stable transformation efficiency in tomato (Micro Tom) and soybean by mitigating cell death [64]. In banana, application of 100 μM melatonin preserved cell health, controlled surface shrinkage and improved the attachment of *Agrobacterium* to the cell surface of banana cells [62]. By reducing ROS levels and suppressing PPO activity, melatonin effectively inhibited the accumulation of total phenolic compounds (TPC) and mitigated tissue browning. These findings highlight the potential of melatonin as a valuable tool to optimize the transformation process, minimize physical and chemical stress on the target tissue, and reduce the potential for tissue injury leading to increasing the success rate of plant regeneration in rough lemon.

Although we tested other antioxidants and osmoprotectants in this study, none of them caused significant improvement for the genetic transformation process compared with melatonin. Melatonin, as an antioxidant and signaling molecule, has been reported to have various beneficial effects on plant growth and stress tolerance [65]. Melatonin also contributes to the plant’s defense against pathogens and pests by activating the plant’s immune system. It enhances the production of defense-related compounds, such as phytoalexins and pathogenesis-related (PR) proteins, to inhibit pathogen growth [66]. Our findings suggested that optimizing genetic transformation of rough lemon following melatonin supplementation refers to multiple factors beyond its antioxidant activity. The precise mechanisms through which melatonin enhances the transformation process in rough lemon may involve additional signaling pathways and molecular processes. Further research is needed to elucidate the specific mechanisms underlying melatonin’s effects on genetic transformation and to explore its potential applications in enhancing plant transformation efficiency and stress tolerance.

The evaluation of transgenic rough lemon lines under NaCl stress revealed their enhanced salt tolerance compared to non-transgenic controls. The localization of the *CcNHX1* transgene in the cell membrane confirms its role in membrane-associated functions and ion transport processes, supporting its potential involvement in salt stress tolerance. The visible damage observed in non-transgenic controls and the reduction in chlorophyll content indicate the detrimental effects of high salt concentration on plant physiological performance. In contrast, the transgenic lines displayed healthy conditions and preserved chlorophyll content, indicating their improved salt tolerance. NHX proteins are membrane proteins present in various membranes of plant cells, including the plasma membrane and endomembrane compartments [67]. NHX proteins facilitate the exchange of sodium (Na^+^) and potassium (K^+^) ions with protons (H^+^) using the proton motive force generated by plasma membrane H^+^ ATPase (P-H^+^ ATPase) and vacuolar H^+^ ATPase (V-H^+^ ATPase) coupled with H^+^ pyrophosphatase (H^+^ PPase) [68]. This mechanism ensures pH and ion homeostasis in plants during normal growth and under stress conditions [69]. Genetically modified models and crop plants expressing NHX proteins from both glycophytes and halophytes have been extensively studied for their ability to enhance tolerance to abiotic stresses, particularly salt and drought stress [70]. In addition to their role in ion homeostasis, NHX proteins are involved in various physiological processes such as cell regulation, osmotic adjustment, turgor maintenance, protein processing and trafficking, microtubule organization, and the development of roots, embryos, and flowers [71–73]. Recent studies using T-DNA insertional mutants and gene knockout approaches have provided further evidence of the importance of NHX proteins in abiotic stress tolerance, as indicated by altered leaf growth, changes in cell size and stomatal functions [74]. In summary, NHX antiporters play a critical role in maintaining cellular homeostasis under abiotic stress conditions in plants [67]. These findings highlight the significance of the *CcNHX1* gene integration and expression in conferring salt stress tolerance to rough lemon. The successful localization of the *CcNHX1* transgene in the cell membrane, combined with the improved performance of transgenic lines under salt stress, suggests that *CcNHX1* plays a crucial role in ion homeostasis and membrane dynamics, contributing to salt stress tolerance in plants.

## Conclusion

Supplementing antioxidants into the tissue culture medium effectively addressed the issues of tissue browning and poor regeneration in rough lemon. Melatonin played a significant role in improving stem regeneration and transformation efficiency. The antioxidant supplementation helped reduce the induction of phenolic compounds and the activity of polyphenol oxidase in rough lemon tissues. Additionally, the study revealed that the *CcNHX1* transgene was localized in the cell membrane. Furthermore, successful integration of the *CcNHX1* gene and subsequent evaluation under salt stress highlighted increased salt tolerance in the transgenic rough lemon lines. As a result, melatonin can be incorporated into an optimized regeneration media to control tissue browning, and genetic modification holds promise for enhancing both biotic and abiotic tolerance in citrus crops.

## Supporting information

S1 Raw images (PDF)
