# Supplementary material for: Melatonin supplementation enhances browning suppression and improves transformation efficiency and regeneration of transgenic rough lemon plants (Citrus × jambhiri)
Source: PLoS One. 2024 Mar 6;19(3):e0294318. doi: 10.1371/journal.pone.0294318 (PMC10917246; doi:10.1371/journal.pone.0294318)

S1 Fig. Original uncropped image of gel image from composite Fig 4. Dotted lines indicate area cropped out from the gel.

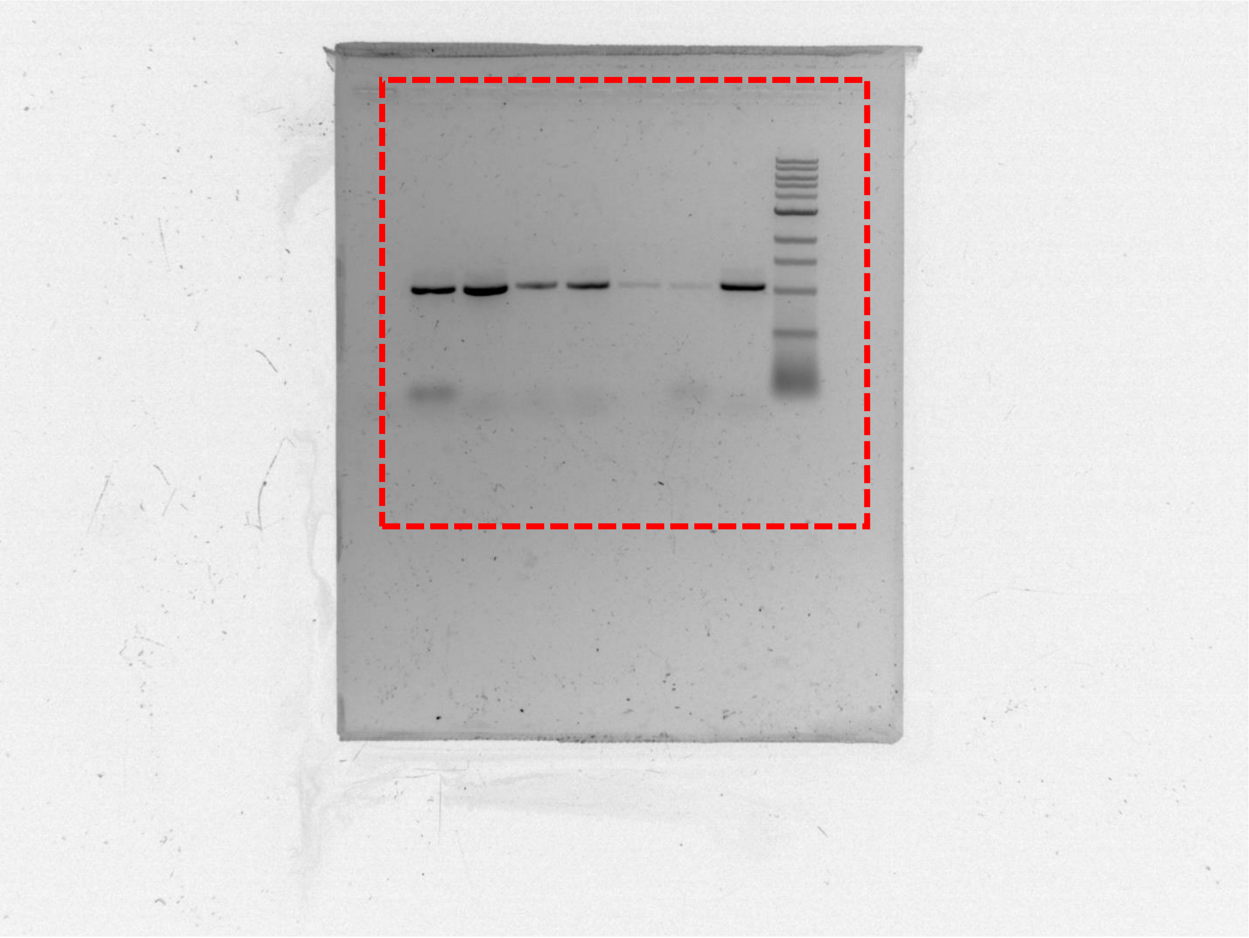

Supplement: S1 Raw images — (PDF) [file pone.0294318.s001.pdf]
